# The bromodomain inhibitor JQ1 triggers growth arrest and apoptosis in testicular germ cell tumours *in vitro* and *in vivo*

**DOI:** 10.1111/jcmm.13059

**Published:** 2016-12-27

**Authors:** Sina Jostes, Daniel Nettersheim, Martin Fellermeyer, Simon Schneider, François Hafezi, Friedemann Honecker, Valerie Schumacher, Matthias Geyer, Glen Kristiansen, Hubert Schorle

**Affiliations:** ^1^ Institute of PathologyDepartment of Developmental PathologyUniversity Medical SchoolBonnGermany; ^2^ Breast and Tumor CenterZeTup SilberturmSt GallenSwitzerland; ^3^ Department of UrologyBoston Children's HospitalBostonMAUSA; ^4^ Department of SurgeryHarvard Medical SchoolBostonMAUSA; ^5^ Institute of Innate ImmunityDepartment of Structural ImmunologyUniversity Medical SchoolBonnGermany; ^6^ Institute of PathologyUniversity Medical SchoolBonnGermany

**Keywords:** bromodomain proteins, JQ1, epigenetic therapy, germ cell cancer, cisplatin resistance, differentiation, pluripotency, romidepsin, HDAC inhibitor, xenografts

## Abstract

Type II testicular germ cell cancers (TGCT) are the most frequently diagnosed tumours in young men (20–40 years) and are classified as seminoma or non‐seminoma. TGCTs are commonly treated by orchiectomy and chemo‐ or radiotherapy. However, a subset of metastatic non‐seminomas (embryonal carcinomas) displays only incomplete remission or relapse and requires novel treatment options. Recent studies have shown effective application of the small‐molecule inhibitor JQ1 in tumour therapy, which interferes with the function of ‘bromodomain and extraterminal (BET)’ proteins. JQ1‐treated TGCT cell lines display up‐regulation of genes indicative for DNA damage and cellular stress response and induce cell cycle arrest. Embryonal carcinoma (EC) cell lines, which presented as JQ1 sensitive, display down‐regulation of pluripotency factors and induction of mesodermal differentiation. In contrast, seminoma‐like TCam‐2 cells tolerated higher JQ1 concentrations and were resistant to differentiation. ECs xenografted *in vivo* showed a reduction in tumour size, proliferation rate and angiogenesis in response to JQ1. Finally, the combination of JQ1 and the histone deacetylase inhibitor romidepsin allowed for lower doses and less frequent application, compared with monotherapy. Thus, we propose that JQ1 in combination with romidepsin may serve as a novel therapeutic option for (mixed) TGCTs.

## Introduction

Approximately 1 of 250 men (age 30–35 years) is diagnosed with TGCT each year 1, 2. These tumours presumably result from a block in primordial germ cell differentiation, giving rise to the precursor lesion termed germ cell neoplasia in situ (GCNIS) 3. GCNIS are found in the spermatogonial niche of the testes near the nursing Sertoli cells and remain dormant until late puberty 2, 4. Between 20 and 45 years of age, they develop into invasive TGCTs, either classified as seminoma or non‐seminoma/EC 5. Currently, TGCTs are treated by orchiectomy and/or additional chemo‐ or radiotherapy 6. However, a subset of 10–30% of metastatic non‐seminomas displays incomplete remission or relapse, due to chemotherapy resistance 2, 7. For these therapy‐resistant tumours, novel treatment options need to be developed. Recently, the cell‐permeable small‐molecule inhibitor (+)‐JQ1 (JQ1) was synthesized, which selectively binds the ‘BET’ family of bromodomain proteins (BRD2, BRD3, BRD4 and BRDT) 8. Bromodomain proteins are epigenetic readers, which bind acetylated histone residues and form complexes with transcription factors to regulate gene expression 9, 10. JQ1 displaces BET proteins from chromatin, thereby interfering with the mRNA elongation of transcribed loci 8.

Interestingly, JQ1 displayed antitumourigenic effects in malignancies such as leukaemia, glioblastoma or prostate cancer models by inhibition of BRD4 11, 12, 13, 14. The BET member BRD4 is a mediator of transcriptional elongation in M/G1 cell cycle transition 15. It recruits the positive transcription elongation factor complex (pTEFb), which activates expression of growth‐promoting and cancer‐associated genes 16. JQ1 was described as the first compound efficiently down‐regulating MYC‐dependent gene expression by abrogating pTEFb‐dependent promoter‐proximal pause release of Polymerase II 16, 17.

In this study, we analysed the molecular and cytotoxic effects of JQ1 application on TGCT cells, Sertoli cells and fibroblasts. JQ1‐mediated bromodomain inhibition resulted in a dose‐dependent increase in apoptosis and G1/G0 arrest in TGCTs. Further, an additive effect on apoptosis was seen when using JQ1 and the histone deacetylase inhibitor romidepsin in combination. We propose that JQ1 alone or in combination with romidepsin may serve as novel therapeutic option for TGCTs.

## Materials and methods

### Cell culture

TGCT cell lines were cultured as described previously 18. In brief, TCam‐2 cells were cultured in RPMI medium (Life Technologies, Darmstadt, Germany). NT2/D1(‐R), 2102EP(‐R) and NCCIT(‐R) cells were cultured in DMEM medium (Life Technologies). Both media were supplemented with 10% FBS (Life Technologies), 1% penicillin/streptomycin (PAN, Aidenbach, Germany) and 200 mM L‐glutamine (PAN). Human adult fibroblast cells (MPAF) were grown in DMEM medium, supplemented with 10% FBS, 1% non‐essential amino acids (NEAA) (PAN), 1% penicillin/streptomycin and 200 mM L‐glutamine. Human Sertoli cells (FS1) were cultured in DMEM medium, supplemented with 20% FBS, 1% NEAA, 1% penicillin/streptomycin and 200 mM L‐glutamine. TCam‐2 cells were a kind gift from Prof. Dr. J. Shipley (Institute of Cancer Research, Sutton, England). NT2/D1, 2102EP and NCCIT were provided by Prof. Dr. L. Looijenga (Erasmus MC, Daniel den Hoed Cancer Center, Josephine Nefkens Institute, Rotterdam, the Netherlands). Cisplatin‐resistant NCCIT (NCCIT‐R), 2102EP (2102EP‐R) and NT2/D1 (NT2/D1‐R) were kindly provided by PD Dr. F. Honecker (Breast and Tumor Center, ZeTup Silberturm, St. Gallen, Switzerland). MPAF were obtained from Dr. M. Peitz (Bonn University, Institute of Reconstructive Neurobiology, Bonn, Germany). FS1 were provided by Dr. V. Schumacher (Boston Children's Hospital, Department of Urology; Harvard Medical School, Department of Surgery, Boston, MA, USA).

### JQ1 and romidepsin treatment *in vitro*

The cells were treated with 100, 250 and 500 nM JQ1 inhibitor (kindly provided by Jay Bradner, Dana‐Farber Cancer Institute, Boston, MA, USA) or 2 and 5 nM romidepsin (Celgene, Signal Pharmaceuticals, LLC, San Diego, CA, USA; MTA ID #CC0488464), respectively. As a negative control, the cells were treated with equal amounts of reconstitution solution (DMSO) only.

### AnnexinV/7AAD and PI‐FACS analysis

AnnexinV/7AAD and PI‐FACS analyses were performed as described before 19. In brief, cells were plated out at a density of 2 × 10^5^ cells/well in a six‐well plate for EC cell lines and 1 × 10^5^ cells/well for TCam‐2, FS1 and MPAF. After indicated time‐points, apoptosis was analysed *via* AnnexinV/7AAD FACS staining, using the PE Annexin V Apoptosis Detection Kit I (BD BioSciences, Heidelberg, Germany). For cell cycle analysis, cells were trypsinized, washed in 1 × PBS and fixed in 100% ice‐cold methanol at −80°C for 2 hrs. After fixation, cells were centrifuged and resuspended in 1 ml PI staining solution (PBS + 2 μl PI (1 mg/ml), +20 μl RNAseA (10 mg/ml)). The cells were analysed (50,000 cells/tube) in a FACS Canto (BD BioSciences).

### XTT assay

For XTT assay, cells were plated out at a density of 3000 cells/well in a 96‐well plate. JQ1/romidepsin was supplemented after 24 hrs. Cells were stained for their viability by XTT after 24/48/72/96 hrs of initial treatment. The XTT assay was performed as described previously 19.

### RNA and protein isolation

For RNA and protein isolation, cells were seeded out at a density of 1 × 10^5^ cells/well in a 6‐well plate prior to initial JQ1/romidepsin treatment. Proteins were isolated using ELISA Lysis buffer (Cell Signaling, Leiden, the Netherlands). The cell lysate was incubated for 10 min. on ice, followed by a 5‐min. centrifugation step at 15,300 × *g* and 4°C. Protein concentrations were determined using the BCA Protein Assay Kit (Thermo Scientific, Rockford, IL, USA). Total RNA was extracted using RNeasy Mini Kit (Qiagen, Hilden, Germany). RNA quality was assessed by photometric measurement of ratios 260/280 nm and 260/230 nm using a NanoDrop photometer (PeqLab, Erlangen, Germany).

### Western blot

Western blot analysis was performed as described elsewhere 19. For detection, the membrane was incubated for 5 min. in 2 ml PierceSuper Signal West Pico chemiluminescent substrate (Thermo Scientific), and the signal was recorded using the Bio‐Rad ChemiDoc™ MP Imaging System (Bio‐Rad, München, Germany). For antibody details, see Table 1. Densitometric quantification of Western blot protein bands was performed with IMAGEJ Software (Wayne Rasband, National Institute of Health, Bethesda, USA). Density values were calculated relative to the loading control (=1).

**Table 1:** Antibodies used in this study

Target	Company	Species	Dilution	Order No.
Antibodies used for Western blot analysis				
BRD2	Sigma‐Aldrich	Rabbit	1:300	HPA042816
BRD3	Abcam	Mouse	1:50	ab50818
BRD4	Active Motif	Rabbit	1:200	39909
Cleaved PARP	Abcam	Rabbit	1:500	ab4830
GDF3	Abcam	Rabbit	1:500	ab38547
HDAC‐1	Santa Cruz	Mouse	1:500	sc81598
LIN28	R&D	Goat	1:1000	AF3757
MYC	Cell Signaling	Rabbit	1:600	5605
OCT3/4	Santa Cruz	Mouse	1:500	sc5279
β‐ACTIN	Sigma‐Aldrich	Mouse	1:50,000	a5441
Anti‐mouse HRP	Invitrogen	Rabbit	1:1000	61‐0120
Anti‐rabbit HRP	Invitrogen	Goat	1:2000	65‐6120
Anti‐goat HRP	Invitrogen	Rabbit	1:2000	61‐1620
Antibodies used for IHC Staining				
Target	Company	Species	Dilution	Order No.
CD31	PECAM	Rat	1:25	SZ31
Ki67	Dako	Mouse	1:100	MIB‐1

### Quantitative real‐time RT‐PCR

Quantitative real‐time RT‐PCR was performed as described previously 20. In brief, cDNA was synthesized using Maxima First Strand cDNA synthesis Kit (Thermo Scientific). For qRT‐PCR, 8.33 ng of cDNA was run in technical triplicates with Maxima SYBR Green qPCR Master Mix (Fermentas, St. Leon‐Rot, Germany). Primer sequences are listed in Table 2 (Table 2). qRT‐PCR was performed using the ViiA™ 7 Real‐Time PCR System (Life Technologies). Quantitative values were obtained from the Ct. *GAPDH* was used as housekeeping gene and for data normalization.

**Table 2:** Oligonucleotides used in this study

Gene	Forward primer	Reverse primer
Primer sequences		
*ATF3*	AAGAACGAGAAGCAGCATTTGAT	TTCTGAGCCCGGACAATACAC
*BRD2*	CTACGTAAAGAAACCCCGGAAG	GCTTTTTCTCCAAAGCCAGTT
*BRD3*	CCTCAGGGAGATGCTATCCA	ATGTCGTGGTAGTCGTGCAG
*BRD4*	AGCAGCAACAGCAATGCTGAG	GCTTGCACTTGTCCTCTTCC
*BRDT*	GCTCGGACACAGGAACTCATACG	CCACCATTGCTTCTCTCCTCCTC
*CDKN1C*	GCGGCGATCAAGAAGCTGT	GCTTGGCGAAGAAATCGGAGA
*MYC*	CGTCTCCACACATCAGCACAA	CACTGTCCAACTTGACCCTCTTG
*DNMT3B*	CCTGCTGAATTACTCACGCCCC	GTCTGTGTAGTGCACAGGAAAGC
*DUSP1*	GTACATCAAGTCCATCTGAC	GGTTCTTCTAGGAGTAGACA
*GADD45B*	GTCGGCCAAGTTGATGAAT	CACGATGTTGATGTCGTTGT
*GAPDH*	TGCCAAATATGATGACATCAAGAA	GGAGTGGGTGTCGCTGTTG
*GDF3*	CAGGAGGAAGCTTGGGAAAT	TGCTAGGTAAAGGAGCTGGG
*HAND1*	AATCCTCTTCTCGACTGGGC	TGAACTCAAGAAGGCGGATG
*ID2*	TCAGCCTGCATCACCAGAGA	CTGCAAGGACAGGATGCTGATA
*LIN28*	TGTAAGTGGTTCAACGTGCG	TGTAAGTGGTTCAACGTGCG
*POU5F1*	GGGAGATTGATAACTGGTGTGTT	GTGTATATCCCAGGGTGATCCTC
*RHOB*	GGGACAGAAGTGCTTCACCT	CGACGTCATTCTCATGTGCT
*THY1*	ATCGCTCTCCTGCTAACAGTC	CTCGTACTGGATGGGTGAACT

### Illumina HumanHT‐12 v4 expression array

Illumina expression microarray analysis was performed as described elsewhere 20. In brief, the cell lines were analysed after 24 and 72 hrs of JQ1 treatment (100 nM) and DMSO as solvent control. Total RNA was extracted and RNA quality was assessed by gel electrophoresis in a BioAnalyser 2100 (Agilent Technologies, Santa Clara, CA, USA). Samples were processed on an Illumina Human HT‐12 v4 Bead Chip (Illumina, San Diego, CA, USA), which was performed at the Institute for Human Genetics, Bonn, Germany. Bioinformatic analysis and data normalization were performed by Andrea Hofman (Institute for Human Genetics, Bonn, Germany). Microarray data sets are publically available *via* GEO: GSE87477 (ncbi.nlm.nih.gov/geo/). Venn diagrams were calculated using Venny (http://bioinfogp.cnb.csic.es/tools/venny/) 21. Protein interaction networks were predicted using STRING analyses (http://string-db.org/) 22.

### *In vivo* xenograft studies

Xenotransplantation was performed as described previously 23. In brief, 1 × 10^7^ cells were resuspended in 500 μl of 4°C cold Matrigel (BD) and injected into the flank of CD1^nu/nu^ mice. Tumours were then grown for 2 weeks and subsequently treated with JQ1 or romidepsin.

### JQ1 and romidepsin treatment *in vivo*

Ten per cent (2‐Hydroxypropyl)‐β‐cyclodextrin (HP‐β‐CD) solution was used as a vehicle, which improves drug solubility (Sigma‐Aldrich, Munich, Germany) 24. The concentrated stock of JQ1 in DMSO was diluted 1/10 in 10% HP‐β‐CD to obtain a final concentration of 50 mg/ml. Romidepsin was added 1/100 to achieve a final concentration of 0.5 mg/ml. For JQ1 treatment, the animals were dosed 50 mg/kg, 5 days/week, i.p. For combination treatment, the animals were dosed 50 mg/kg JQ1 + 0.5 mg/kg romidepsin 3 days/week, i.p. Tumour growth was measured everyday using a calliper.

### Immunohistochemistry

Immunohistochemistry was performed as described previously 20, 25. Tumours were dissected, fixed in 4% formalin at 4°C overnight and processed in paraffin wax. Staining was performed semiautomatically in the Autostainer 480 S (Medac, Hamburg, Germany). For antibody details, see Table 1. Stainings were quantified from three individual tumours (*n* = 3). Significance was calculated using two‐tailed Student's *t*‐test.

## Results

### TGCT cell lines express the BET members BRD2, BRD3 and BRD4

**Figure 1:**
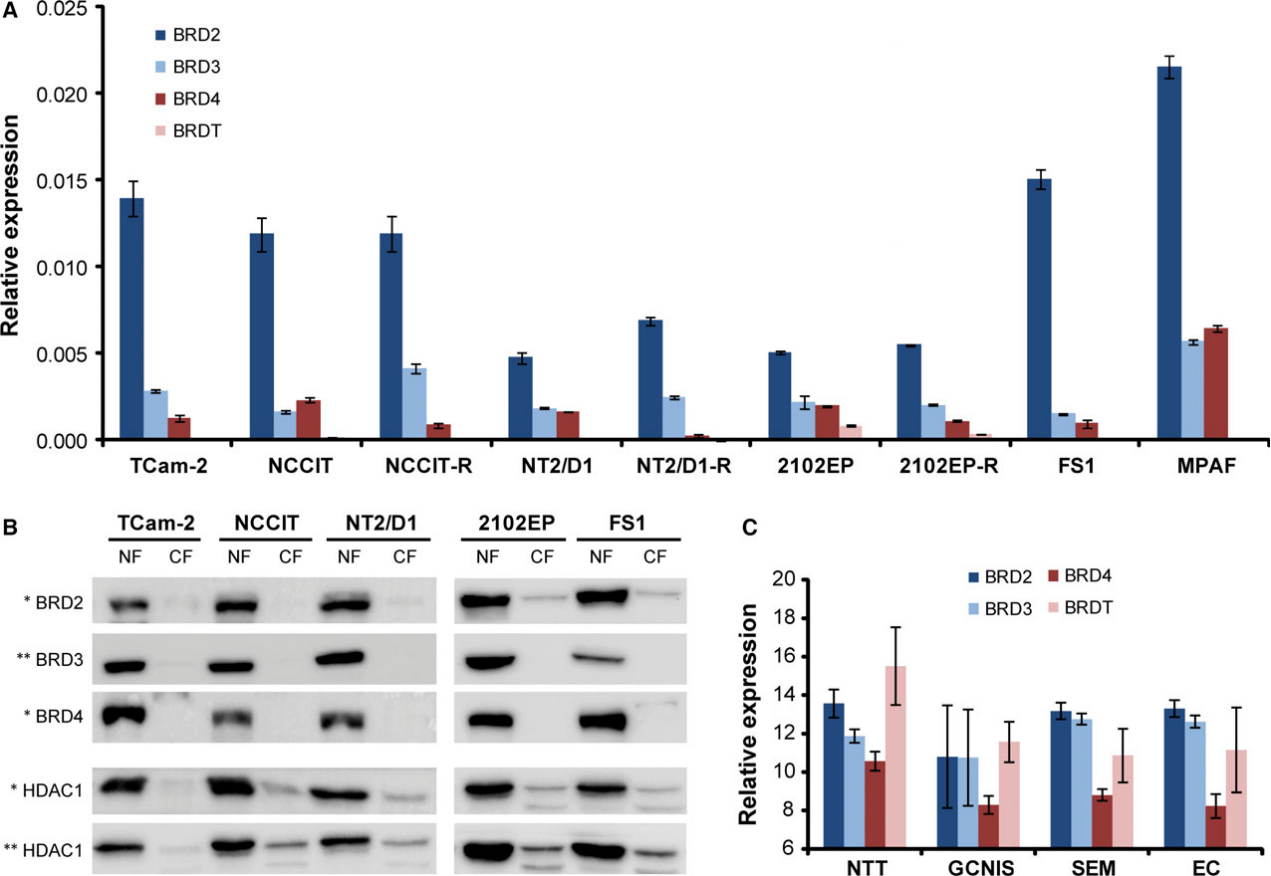
(**A**) *BRD2, BRD3, BRD4* and *BRDT* expressions in human TGCT cell lines (TCam‐2, NCCIT(‐R), NT2/D1(‐R), 2102EP(‐R)), Sertoli cells (FS1) and human fibroblasts (MPAF) as determined by qRT‐PCR. (**B**) BRD2, BRD3 and BRD4 protein levels in human TGCT cell lines and Sertoli cells as found by Western blot analysis. (**C**) *BRD2, BRD3, BRD4* and *BRDT* expression in normal testis tissues (NTT), GCNIS, seminomas (SEM) and embryonal carcinomas (EC) as determined by expression microarray analysis. Standard deviations, calculated by two‐tailed Student's *t*‐test, are given above each bar.

JQ1 is a small‐molecule inhibitor that blocks the binding pocket of bromodomain proteins for acetylated lysine residues of histones, thus interfering with the cellular transcriptional machinery 16. Different studies have demonstrated its potential use in cancer therapy; however, nothing is known so far about the efficacy of JQ1 treatment in TGCTs. First, we determined expression levels of *BRD2*,* BRD3*,* BRD4* and *BRDT* by qRT‐PCR. We utilized three different EC cell lines (NCCIT, NT2/D1, 2102EP) and their cisplatin‐resistant subclones (NCCIT‐R, NT2/D1‐R, 2102EP‐R) as well as the seminoma cell line TCam‐2. As proxies for somatic cells and cells of the testis microenvironment, we included a human fibroblast (MPAF) and Sertoli cell line (FS1) in our analysis. All cell lines showed highest mRNA expression levels of *BRD2* (Fig. 1A). Levels of *BRD3* and *BRD4* were lower, while expression of *BRDT* was very weak to absent (Fig. 1A). The cisplatin‐resistant subclones display comparable levels of *BRD2, BRD3* and *BRDT* as the parental cell lines (Fig. 1A). *BRD4* mRNA levels appeared slightly lower in cisplatin‐resistant cell lines (Fig. 1A). By meta‐analysing previously published microarray data, we confirmed expression levels of *BRD2, BRD3, BRD4* and *BRDT* in TCam‐2, NCCIT, 2102EP, FS1 and MPAF (Fig. S1A) 19. Additionally, we performed Western blot analysis to compare BRD2, BRD3 and BRD4 protein levels to RNA expression. We detected BRD2, BRD3 and BRD4 in the nuclei of TCam‐2, NCCIT, NT2/D1, 2102EP and FS1 Sertoli cells (Fig. 1B). Interestingly, quantitation of protein bands relative to HDAC1 levels revealed that there is no correlation between RNA expression and protein levels in all cell lines analysed (Fig. 1B). The differences between RNA and protein levels might point at differences in regulation of transcription, RNA / protein half‐life or RNA‐protein‐turn‐over between the BRD members. Nevertheless, in all TGCT cell lines and Sertoli cells, BRD2, BRD3 and BRD4 protein levels were detected.

Next, we used previously published microarray data to analyse expression of *BRD2, BRD3, BRD4* and *BRDT* in TGCT tissues 26. Expression levels were measured in normal testis tissues, GCNIS lesions, seminomas and ECs and presented as highly similar to the *in vitro* expression data (Fig. 1C *versus* A). In normal testis tissue, high *BRDT* expression levels were detected, while TGCT tissues showed significantly lower levels. We hypothesize that the expression of *BRDT* in TGCT tissues reflects the presence of *BRDT* positive germ cells (Fig. 1C) 27, 28. We conclude that in TGCTs, *BRD2*,* BRD*3 and *BRD*4 are expressed, and thus, application of JQ1, which blocks BET function, may affect growth and survival of TGCTs as well as normal gonadal cells.

### JQ1 induces apoptosis and cell cycle arrest in TGCT cell lines

**Figure 2:**
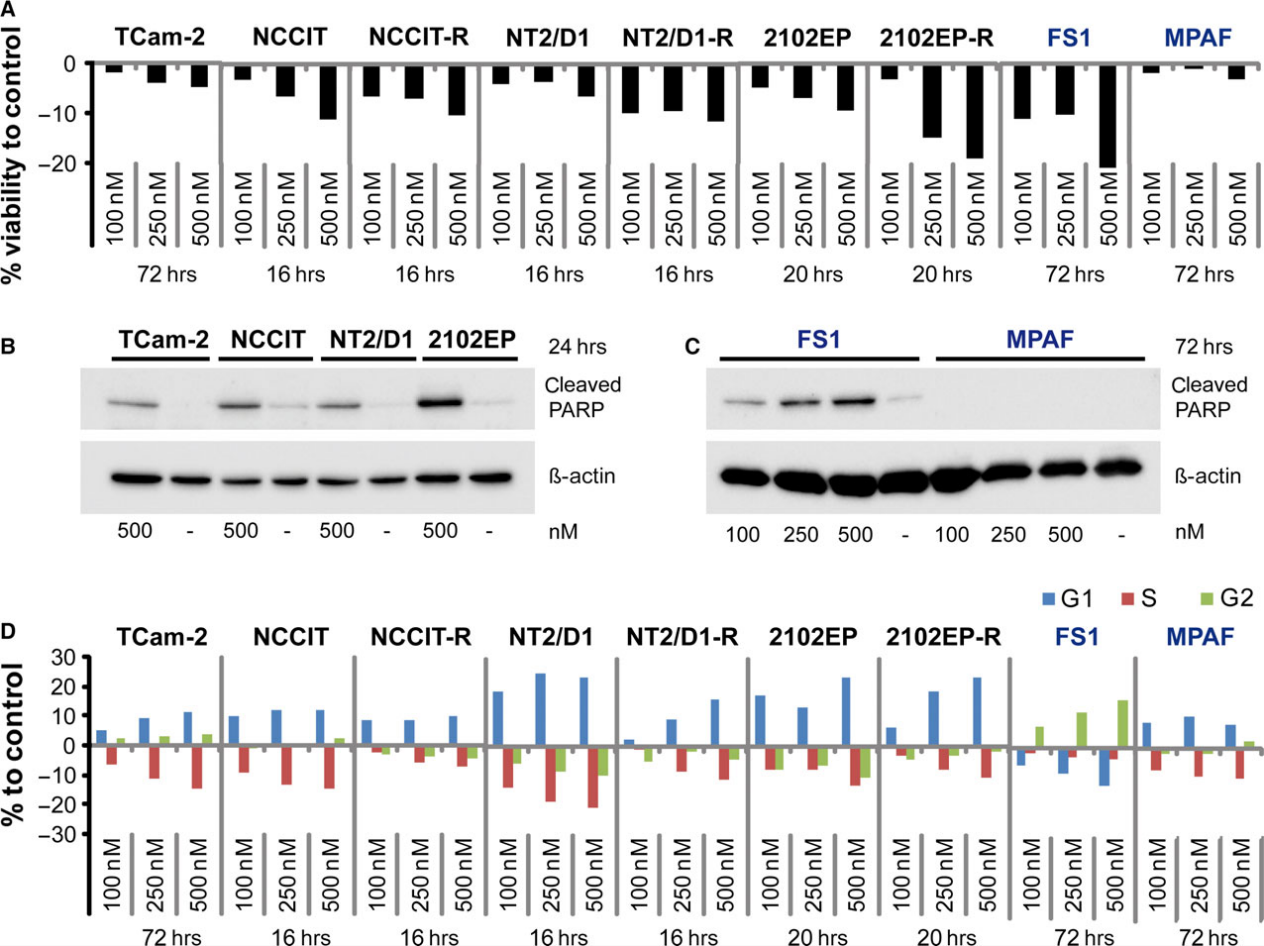
(**A**) AnnexinV/7AAD FACS analysis of apoptosis in TGCT cell lines (TCam‐2, NCCIT(‐R), NT2/D1(‐R), 2102EP(‐R)), Sertoli cells (FS1) and human fibroblasts (MPAF) after JQ1 treatment. (**B**,** C**) Western blot analysis of cleaved PARP in 500 nM JQ1‐treated TGCT cell lines (TCam‐2, NCCIT, NT2/D1, 2102EP) (**B**), Sertoli cells (FS1) and fibroblasts (MPAF) (**C**). (**D**) PI‐FACS analysis of cell cycle distribution of TGCT cell lines, Sertoli cells (FS1) and fibroblasts (MPAF) after JQ1 treatment. Standard deviations, calculated by two‐tailed Student's *t*‐test, are given above each bar.

Next, we treated TGCT and control cell lines with 100–500 nM JQ1 and used AnnexinV/7AAD FACS analysis to measure induction of apoptosis. In all TGCT cell lines, JQ1 markedly increased the fraction of apoptotic cells in a dose‐dependent manner (Fig. 2A, Fig. S2A). After 16 hrs of treatment, the lowest JQ1 dose (100 nM) initiated apoptosis in the EC cell lines NCCIT(‐R) and NT2/D1(‐R). The nullipotent EC cell lines 2102EP(‐R) displayed apoptosis later after 20 hrs. Interestingly, two (2102EP‐R, NT2/D1‐R) of three cisplatin‐resistant subclones displayed a higher sensitivity to JQ1‐induced apoptosis compared with the parental EC cell lines (Fig. 2A). TCam‐2 cells did not display apoptosis before 72 hrs at all three concentrations (100, 250 and 500 nM), suggesting a reduced sensitivity towards JQ1 (Fig. 2A, Fig. S2B). Indeed, 100 nM JQ1 was tolerated by TCam‐2 cells over a time period of 16 weeks (Fig. S2C), but 500 and 250 nM JQ1 induced apoptosis in TCam‐2 cells after 8 and 15 days, respectively (Fig. S2B). Notably, the Sertoli cell line FS1 showed strong induction of apoptosis after 72 hrs of JQ1 treatment, indicating adverse effects of JQ1 treatment on the microenvironment of the testes, that is Sertoli cells (Fig. 2A, Fig. S3A). In contrast, cell viability of fibroblasts remained unchanged (Fig. 2A, Fig. S3A). To confirm the induction of apoptosis, cleavage of PARP was analysed by immunoblotting in JQ1‐treated TGCT and control cell lines. Treatment with 500 nM JQ1 resulted in PARP cleavage in TGCT cell lines and Sertoli cells after 24 and 72 hrs, respectively (Fig. 2B and C). This effect was not detectable in fibroblasts, further stressing the point that fibroblasts are more resistant to JQ1 (Fig. 2C).

In glioblastoma, leukaemia and medulloblastoma cells, JQ1 treatment induced G1 cell cycle arrest 12, 17, 29. Here, we used PI‐FACS to analyse cell cycle distribution in JQ1‐treated TGCT cell lines. As expected, all TGCT cell lines displayed a pronounced decrease of cells in S‐phase and accumulation of cells in G1/G0 phase (Fig. 2D). Of note, even TCam‐2 cells treated for 16 weeks with 100 nM JQ1 enriched in G1/G0‐phase (Fig. S3B). G1/G0 arrest was also seen in fibroblasts, while Sertoli cells display accumulation of cells in G2/M‐phase, indicative for a G2/M cell cycle arrest (Fig. 2D). We conclude that in ECs JQ1 treatment leads to induction of apoptosis at concentrations ≥100 nM, while in seminomas apoptosis is induced at concentrations ≥250 nM. Also, JQ1 treatment leads to accumulation of TGCT cells in G1/G0 phase at concentrations ≥100 nM. However, JQ1 treatment is also reducing cell viability and interfering with cell cycle progression of Sertoli cells, indicating an adverse effect on the testis microenvironment. In contrast, human fibroblasts are resistant to the pro‐apoptotic effects of JQ1 and display cell cycle arrest only.

### JQ1 up‐regulates DNA damage and cellular stress sensors and down‐regulates pluripotency factors in TGCT cell lines

Next, we analysed the changes in gene expression after JQ1 treatment. TGCT cell lines (NCCIT and TCam‐2), human Sertoli cells (FS1) and fibroblasts (MPAF) were treated with 100 nM JQ1 and subjected to expression microarray analysis (Data S1A‐D). Venn diagrams summarize the numbers of deregulated genes in all cell lines analysed (Data S1A‐D), indicating a total number of 329 deregulated genes in TCam‐2 and 601 genes in NCCIT (Data S1A and B). JQ1‐resistant fibroblasts displayed differential expression of 180 genes, while JQ1‐sensitive FS1 cells showed deregulation of 48 genes only (Data S1C and D).

**Figure 3:**
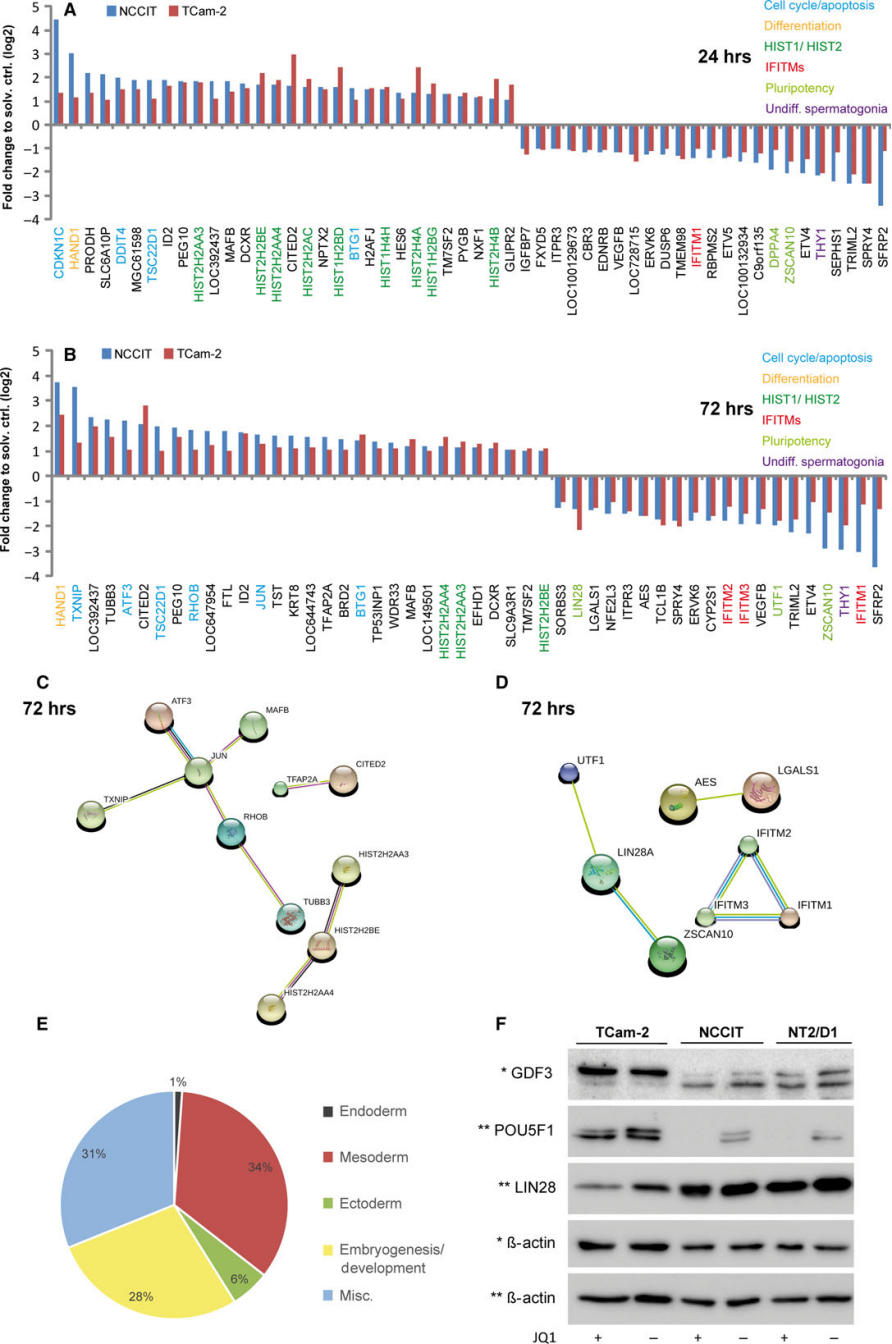
(**A**,** B**) Microarray analysis of JQ1‐treated TCam‐2 and NCCIT cells after 24 (**A**) and 72 (**B**) hrs normalized to solvent controls. (**C**,** D**) STRING interaction networks of commonly up‐ (**C**) and down‐regulated (**D**) genes in TGCT cell lines after 72 hrs. (**E**) Pie chart summarizing the numbers of enriched biological processes among the up‐regulated genes in NCCIT cells after 72 hrs of JQ1 treatment. Data were sorted for the categories ‘endodermal’, ‘mesodermal’ and ‘ectodermal differentiation’, ‘embryogenesis/development’ and ‘miscellaneous’. (**F**) Western blot analysis showing protein levels of PRDM14, POU5F1 and LIN28 in JQ1‐treated TGCT cell lines.

Comparing the group of tumour cell lines (TCam‐2, NCCIT) to somatic cells (MPAF, FS1), revealed no commonly deregulated genes (Data S1E), indicating a different response towards JQ1 treatment. However, all four cell lines showed up‐regulation of genes of the *HIST1* and *HIST2* clusters, which encode for building blocks of histone molecules 30 (Fig. 3A and B; green labels, Data S1A–D; green labels), an effect that was already demonstrated in previous publications 16, 17, 31. We then sorted for commonly deregulated genes in TCam‐2 and NCCIT only and found several genes up‐regulated after 24 hrs, classified as ‘early effects’, that is strong up‐regulation of the cell cycle arrest‐ and apoptosis‐associated genes *CDKN1C*,* DDIT4, TSC22D1* and *BTG1* (Fig. 3A, blue labels) and induction of the mesodermal differentiation marker *HAND1* (Fig. 3A, yellow labels) 32, 33, 34, 35, 36. We utilized STRING algorithm to predict protein–protein interactions among the commonly early up‐regulated genes. There, interaction of the HIST1 and HIST2 cluster members was predicted (Fig. S4A).

Among the ‘late effects’ (72 hrs), we found up‐regulation of the antiproliferative markers *TXNIP, ATF3*,* RHOB* and *JUN* 37, 38, 39 (Fig. 3B, blue labels). Additionally, we still found up‐regulation of the markers *HAND1*,* TSC22D1* and *BTG1* (Fig. 3B, yellow and blue labels). STRING algorithm predicted a network consisting of TXNIP, ATF3, MAFB, RHOB, JUN and TUBB3, suggesting that these factors interact in response to JQ1 (Fig. 3C). Up‐regulation of *ATF3*,* RHOB* and *HAND1* after 72 hrs was confirmed by qRT‐PCR analysis in TCam‐2, NCCIT and additionally NT2/D1 (Fig. S5A). Of note, the nullipotent EC cell line 2102EP did not display up‐regulation of these markers at similar concentrations (100 nM), but showed up‐regulation of *ATF3*,* RHOB* and *HAND1* at JQ1 concentrations starting from 750 nM, indicating a different dose‐response of the TGCT cell lines TCam‐2, NCCIT and NT2/D1 compared to nullipotent 2102EP cells (Fig. S5B).

Among the down‐regulated genes, we found *THY1*, a marker for undifferentiated spermatogonia, which is suppressed after 24 and 72 hrs of JQ1 treatment (Fig. 3A and B, purple labels) 40, 41. By qRT‐PCR analysis, down‐regulation of *THY1* was confirmed in TCam‐2, NCCIT, NT2/D1 and 2102EP (only at concentrations ≥750 nM), but was absent in FS1 and MPAF (Fig. S5A and B). Similarly, treatment of TGCT cell lines with JQ1 for 72 hrs resulted in down‐regulation of three members of ‘Interferon‐induced Transmembrane Protein’ family (*IFITM1 ‐ 3)*, which were predicted to interact by STRING (Fig. 3A and B, red labels; Fig. 3D). In mice, *Ifitm1‐3* encode for cell surface proteins that may confer distinct guidance cues for migrating primordial germ cells 42, 43. Thus, down‐regulation of *THY1* and *IFITM1‐3* may suggest a loss of germ cell‐like characteristics and the induction of differentiation.

Additionally, we detected down‐regulation of a set of pluripotency‐associated factors (*ZSCAN10, UTF1*,* LIN28*) (Fig. 3A and B, light green labels) 44, 45, 46, 47, 48. Of these, LIN28A, ZSCAN10 and UTF1 are predicted to interact by STRING (Fig. 3D). To analyse the effects of JQ1 on the pluripotency circuitry of TGCTs in more detail, we sorted for genes specifically deregulated in the pluripotent EC cell line NCCIT and detected strong down‐regulation of *GDF3*,* POU5F1*,* NANOG* and *DNMT3B* (in addition to already detected *ZSCAN10*,* UTF1* and *LIN28)* (Data S1B) 20, 49, 50. A GeneTrail‐based ‘Gene Ontology’ analysis of all genes up‐regulated (fold change ≥Log_2_1.0) in NCCIT cells demonstrated that down‐regulation of pluripotency factors coincided with up‐regulation of marker genes indicative for differentiation into all three germ layers (mainly mesoderm) (Fig. 3E, Data S1F). Further*,* qRT‐PCR analysis verified down‐regulation of *LIN28, POU5F1, GDF3* and *DNMT3B* in NCCIT, NT2/D1 and 2102EP (again only at concentrations ≥750 nM) (Fig. S5A and B). To validate these findings on protein level, we measured GDF3, POU5F1 and LIN28 expression in JQ1‐treated TGCT cell lines by immunoblotting. In line with the qRT‐PCR data, GDF3, POU5F1 and LIN28 (only slightly) are down‐regulated in NCCIT and NT2/D1 (Fig. 3F). In TCam‐2, we only found down‐regulation of LIN28 protein after 72 hrs of JQ1 treatment (Fig. 3F). These findings suggest that JQ1 represses the pluripotency circuitry in TGCT cell lines, resulting mainly in differentiation into mesoderm.

Of note, BRD4 (and co‐factors) activates *MYC* transcription 16, 17. Thus, JQ1‐based interference with BRD4 function should lead to reduction of MYC levels, as previously described 16, 17. However, increasing evidence suggests that some cancer types escape JQ1‐mediated repression of *MYC* 51, 52, 53. In this study, we found weak (NCCIT, NT2/D1, 2102EP) to strong (TCam‐2) up‐regulation of *MYC* transcript levels 24–72 hrs after JQ1 application (Fig. S6A). In contrast to the mRNA data, MYC protein levels remained unaffected (Fig. S6B). Unexpectedly, TCam‐2, which tolerated higher levels of JQ1, displayed reduced levels of MYC protein at 500 nM (Fig. S6B). So, the role of MYC in the response of TGCT cell lines to JQ1 treatment remains elusive.

In summary, we were able to show that JQ1 treatment of TGCT cell lines *in vitro* results in up‐regulation of DNA damage and stress sensors in TCam‐2, NCCIT, NT2/D1 and 2102EP cells. Further, in EC cell lines, we demonstrated down‐regulation of pluripotency‐associated genes and strong induction of germ‐layer differentiation markers. These effects were not observed in human fibroblasts or Sertoli cells.

### JQ1 treatment reduces tumour burden of mice with xenografted EC cells

**Figure 4:**
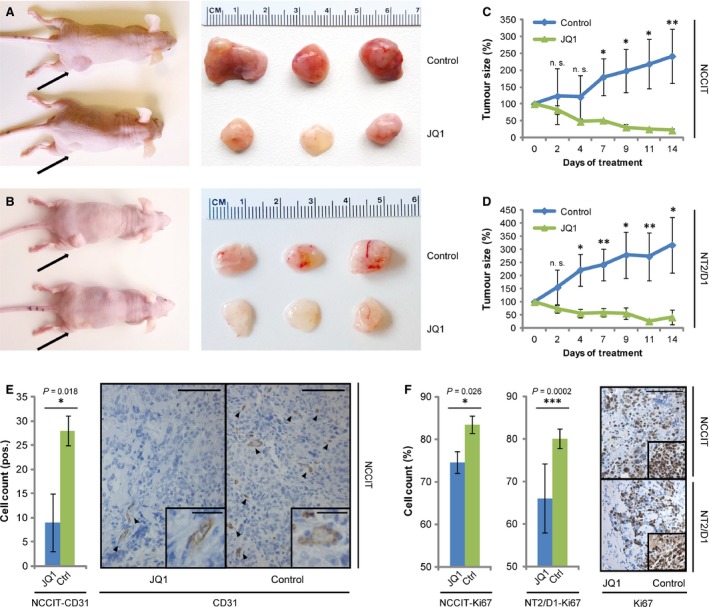
(**A**,** B**) Macroscopic appearance of JQ1‐ and solvent‐treated NCCIT‐ (**A**) and NT2/D1‐(**B**) xenografted tumours *in vivo* (left) and *ex vivo* (right). (**C**,** D**) Growth curve of NCCIT‐ (**C**) and NT2/D1‐(**D**) xenografted tumours treated with JQ1 (50 mg/kg, 5 days/week) or the solvent. (**E**) IHC staining of CD31 in NCCIT‐xenografted tumour tissues treated with JQ1 or the solvent. Left: quantification of IHC data, right: representative image of CD31 staining. Scale bar: 100 μm. Inlays show zoom‐in of CD31^+^ cells. Scale bar: 25 μm. (**F**) IHC staining of Ki67 in NCCIT‐ and NT2/D1‐xenografted tumour tissues treated with JQ1 or the solvent. Left: quantitation of IHC data, right: representative image of Ki67 staining. Scale bar: 100 μm. Standard deviations and *P*‐values (*P*), calculated by two‐tailed Student's *t*‐test, are given above each bar. *P < 0.05, **P < 0.005, ***P < 0.0005, n.s.: not significant.

To evaluate the therapeutic efficacy of JQ1 application *in vivo*, we xenografted two different EC cell lines (NCCIT, NT2/D1) into the flank of nude mice. After 2 weeks of initial tumour growth, we treated these mice 5 days/week with 50 mg/kg JQ1 i.p. for 2 weeks. After 1 week, the xenografted animals showed a significant reduction in tumour burden after JQ1 treatment compared with animals treated with solvent only (Fig. 4A–D). Additionally, JQ1‐treated tumours displayed a pale and opaque texture and showed a reduced infiltration of blood vessels, suggesting reduced angiogenesis (Fig. 4A and B). In line, previous studies demonstrated suppressed tumour angiogenesis after JQ1 treatment in *in vivo* models of childhood sarcoma 54. Immunohistochemistry verified diminished angiogenesis in JQ1‐treated NCCIT tumours by detecting significantly reduced CD31 staining, a marker for endothelial cells 55 (Fig. 4E). Additionally, immunohistochemical staining demonstrated a significant reduction of Ki67‐positive cells in JQ1‐treated tumours, confirming reduced cellular proliferation (Fig. 4F). So, EC xenografts are affected by JQ1 in two ways, they are restricted in tumour growth and neo‐angiogenesis.

### Combination treatment with JQ1 and romidepsin effectively reduces cell viability of seminoma and EC components

**Figure 5:**
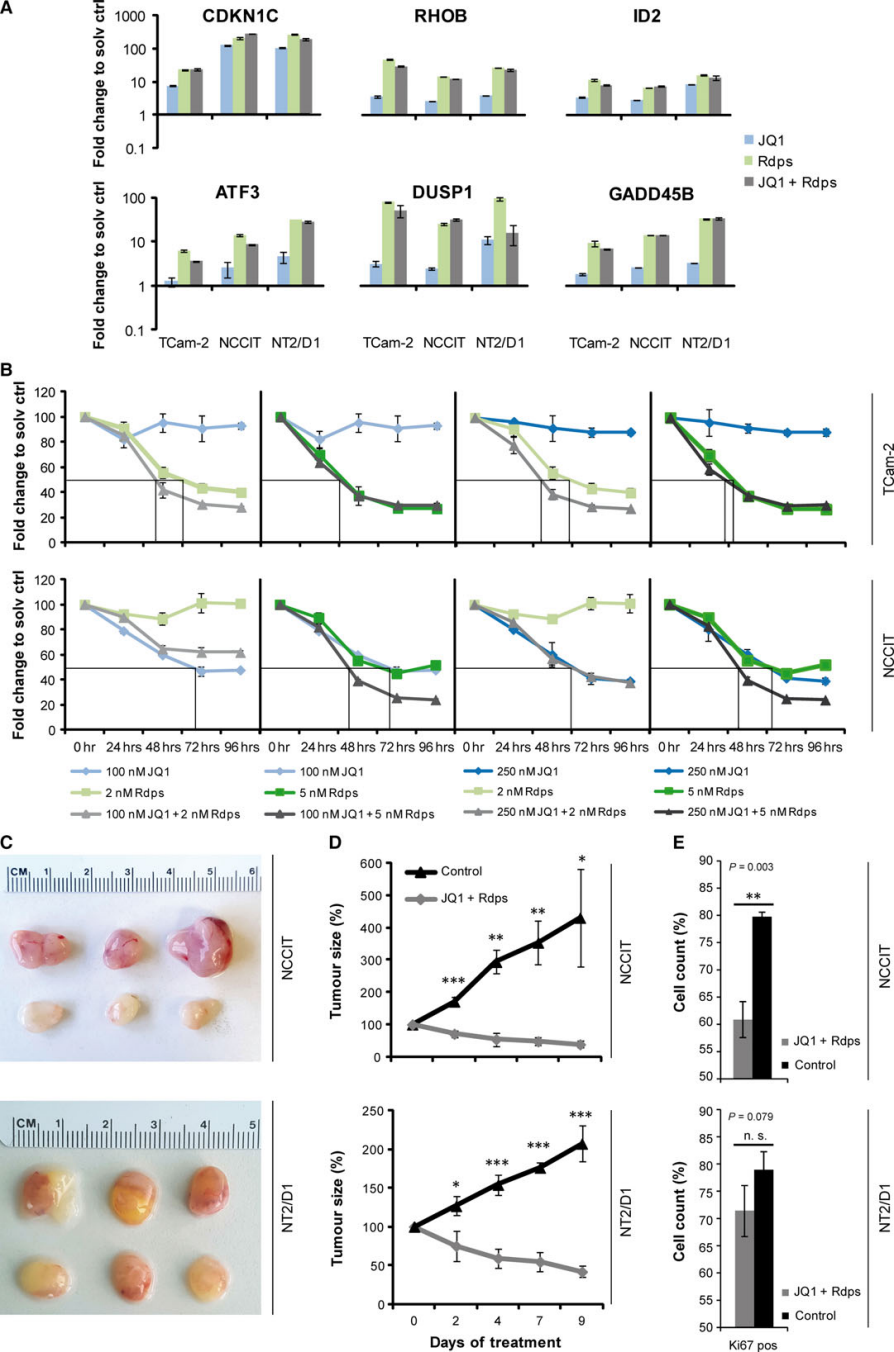
(**A**) qRT‐PCR analysis of indicated genes deregulated after JQ1 (100 nM), romidepsin (5 nM) and JQ1 + romidepsin (100 + 5 nM) treatment in TGCT cell lines after 24 hrs normalized to solvent controls. (**B**) Measurement of cell viability of NCCIT and NT2/D1 cells after a single application of JQ1 and/or romidepsin, as assessed by XTT assay over 96 hrs. (**C**) Macroscopical appearance of JQ1 + romidepsin (50 + 0.5 mg/kg) or solvent‐treated NCCIT‐ and NT2/D1‐xenograft tumours *ex vivo*. (**D**) Tumour growth of NCCIT‐ and NT2/D1 xenografts treated with JQ1 + romidepsin (50 + 0.5 mg/kg) or the solvent. (**E**) IHC of Ki67 in NCCIT‐ or NT2/D1‐xenografted tumour tissues treated with JQ1 + romidepsin (50 + 0.5 mg/kg) or the solvent. Standard deviations and *P*‐values (*P*), calculated by two‐tailed Student's *t*‐test, are given above each bar. *P < 0.05, **P < 0.005, ***P < 0.0005, n.s.: not significant.

JQ1 treatment induced *CDKN1C, RHOB, ID2* and *ATF3* expression in TCam‐2, NCCIT and NT2/D1, as determined by microarray (Data S1A and B) or qRT‐PCR analysis (Fig. 5A). Interestingly, we published recently that a similar up‐regulation of *RHOB, ATF3, CDKN1C* and *ID2* is detected in TCam‐2, NCCIT and NT2/D1 upon treatment with the HDAC inhibitor romidepsin (Fig. 5A) 19. Thus, we asked, whether further romidepsin effector genes were up‐regulated in JQ1‐treated TGCT cell lines. Indeed, similar to romidepsin‐treated TGCT cell lines, we demonstrated up‐regulation of *DUSP1* and *GADD45B* after JQ1 (Fig. 5A). Of note, expression of these markers was not further increased after combinatorial treatment with both epigenetic drugs (Fig. 5A). So, romidepsin and JQ1 treatment induce a similar stress response in TGCT cell lines.

In a murine lymphoma model, combined HDAC and BET inhibitor treatment led to enhanced apoptosis, improving the overall survival of lymphoma‐bearing mice compared to mice treated with the respective monotherapies 56. Further, in human breast cancer cell lines, JQ1 and the HDAC inhibitor mocetinostats were shown to synergistically reduce cell viability and cell cycle progression 57. Thus, we asked whether a combination therapy would increase the pro‐apoptotic effects of JQ1 or romidepsin monotherapy in TGCT *in vitro*. Therefore, we treated TGCT cell lines once with either JQ1 (100 and 250 nM) or romidepsin (2 and 5 nM) or both drugs in combination prior to a 96‐hrs incubation period. Cell viability was assessed by XTT assay every 24 hrs relative to solvent‐treated control cells (Fig. 5B). We found that TCam‐2 were sensitive to low‐dose romidepsin treatment (2 nM), but resistant to a single application of JQ1 (100 or 250 nM) (Fig. 5B). In contrast, the cytotoxicity of low‐dose romidepsin application (2 nM) in TCam‐2 was enhanced by additional application of 100 or 250 nM JQ1 (Fig. 5B). NCCIT cells were sensitive to JQ1 treatment (100 or 250 nM), while a low dose of romidepsin (2 nM) had only mild effects on viability (Fig. 5B). Only application of 5 nM romidepsin plus 100 or 250 nM JQ1 strongly reduced viability of NCCIT cells (Fig. 5B). Hence, we found that compared to EC cell lines seminoma‐like TCam‐2 are more sensitive to romidepsin, while being more resistant to JQ1. Vice versa, compared to TCam‐2, EC cell lines are sensitive to JQ1, while being more resistant to romidepsin. Thus, a subject diagnosed with a mixed TGCT, including seminoma and EC populations, would benefit from a combination of HDAC and BET inhibitors.

We then asked whether this combination of BET and HDAC inhibitors also kills TGCTs *in vivo*. Therefore, NCCIT and NT2/D1 cells were xenografted into the flank of 6‐week‐old nude mice and after 2 weeks of initial tumour growth, mice were treated 3 days/week with 50 mg/kg JQ1 + 0.5 mg/kg romidepsin i.p. for 10 days. The animals show a significant reduction in tumour burden after 2 days (Fig. 5C and D). Importantly, a similar reduction of tumour burden was achieved using a less frequent application of JQ1 + romidepsin (3 times/week) as compared to JQ1‐only treated animals (5 times/week) (Figs 5D *versus* 4C and D). Additionally, romidepsin concentrations used in this *in vivo* experiment (0.5 mg/kg) were considerably lower than in a study published recently (2 mg/kg) 19. This suggests that a combination of both drugs may be administered less frequently and at lower doses, still resulting in a comparable reduction of tumour burden as under monotherapy. Similar to JQ1‐treated NCCIT‐ and NT2/D1‐derived tumours, the JQ1 + romidepsin‐treated tumours displayed reduced infiltration of blood vessels and a pale, opaque morphology (Fig. 5C). Further, immunohistochemical staining showed a reduction in Ki67‐positive cells, suggestive of reduced cell proliferation (Fig. 5D). In conclusion, additive effects of both drugs allow for a less frequent application scheme compared to monotherapy.

## Discussion

The BET protein family has been described as a promising target for epigenetic therapy and led to the development of the putative cancer therapeutic JQ1 16, 58. A short elimination half‐life and a good pharmacokinetic profile provide JQ1 with ideal drug‐like properties 59. JQ1 inhibits the BET member BRD4, which is involved in regulation of genes associated with growth and cell cycle progression; thus, JQ1 was considered as a cancer therapeutic 8.

**Figure 6:**
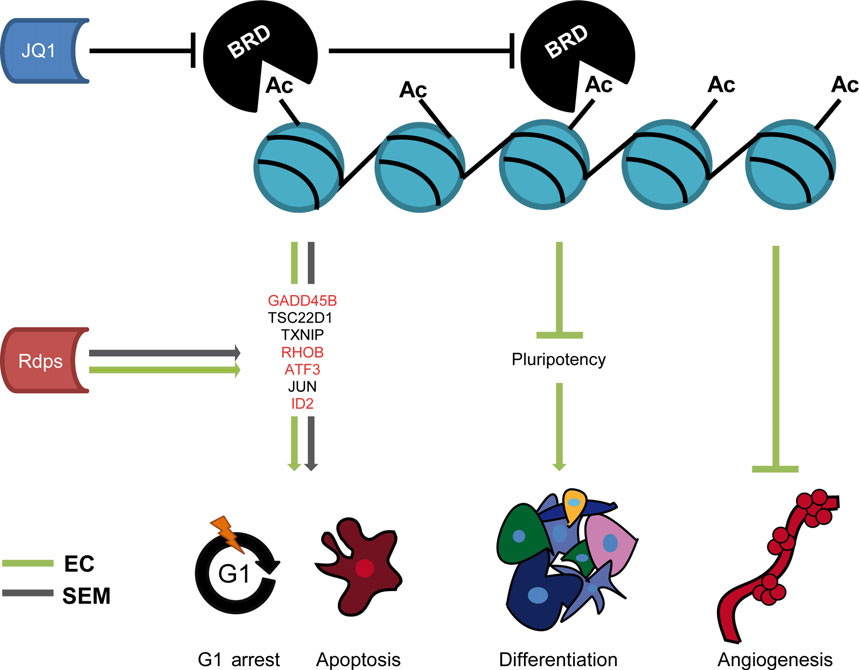
Model summarizing the molecular effects of JQ1 on TGCT cell lines. JQ1‐mediated inhibition of BRD proteins results in a loss of pluripotency‐associated markers in EC cell lines, leading to a teratoma‐like differentiation. Also, JQ1 inhibits tumour angiogenesis in EC cell lines. In seminoma and EC cell lines, JQ1 leads to up‐regulation of DNA damage‐ and stress response genes, which are in parts also induced by the HDAC inhibitor romidepsin (rdps.) (red labelled genes). These genes putatively contribute to G1 cell cycle arrest and apoptosis.

Here, we analysed the molecular effects of JQ1 on TGCT cell lines, while utilizing human fibroblasts and a Sertoli cell line as controls. We found that JQ1 induces DNA damage and stress response genes, putatively contributing to the induction of cell death and cell cycle arrest in the GCNIS/seminoma‐like cell line TCam‐2 and the EC cell lines NCCIT, NT2/D1 and 2102EP (Fig. 6). We further demonstrated that tumours from xenografted EC cells were significantly reduced in size and proliferation rate after JQ1 application. Also, we found a reduced infiltration of blood vessels in JQ1‐treated tumours (Fig. 6).

Interestingly, EC cell lines show robust down‐regulation of pluripotency genes and up‐regulation of differentiation markers, suggesting induction of mesoderm (Fig. 6). There are therapeutic strategies aiming to induce terminal differentiation of tumour cells, rendering them less aggressive 60. In this respect, JQ1 not only induces apoptosis in ECs, but might lead to acquisition of a less aggressive state. In TCam‐2, although down‐regulation of some pluripotency genes was observed, no indications for a differentiation process were found (except up‐regulation of *HAND1*). This is in line with previous findings, demonstrating the ability of TCam‐2 cells to counteract differentiation‐inducing stimuli 61, 62.

Notably, JQ1 concentrations affecting survival of EC cell lines (≥100 nM) are comparable to doses killing VCaP prostate cancer or MCF7 breast cancer cells 11, 63. In contrast, TCam‐2 cells were able to tolerate doses ≤100 nM up to 4 months and showed first signs of apoptosis at doses ≥250 nM. Puissant and Lockwood *et al*. defined an IC50 >1000 nM as JQ1: resistance 31, 64. Thus, both EC and seminoma cells can be described as JQ1 sensitive. However, EC cells presented as highly sensitive to JQ1 treatment in comparison with the more resistant seminoma‐like cell line TCam‐2. It is tempting to speculate whether drug sensitivity of TGCT cell lines goes in hand with the ability to differentiate. In line, 2102EP cells, which are nullipotent and thus not prone to differentiate, are also more resistant to JQ1 than the pluripotent EC cell lines NCCIT and NT2/D1.

In fibroblasts and Sertoli cells, we also observed induction of cell cycle arrest, but apoptosis was restricted to Sertoli cells. Thus, JQ1 affects the cell cycle of non‐transformed cells and the cytotoxicity on Sertoli cells indicates side effects on the testis microenvironment. Although previous studies in mice did not report any adverse effects of JQ1 on cells of the testis epithelium, further *in vivo* data need to explore the effects on human testes cells 59, 65, 66.

Finally, we demonstrate that in TGCT cell lines, JQ1 elicits a similar stress response as the HDAC inhibitor romidepsin, although both drugs act by a different molecular mechanism (romidepsin: inhibits HDACs, leads to histone hyperacetylation; JQ1: inhibits BRD proteins, interferes with reading the histone code) (Fig. 6). Furthermore, the pro‐apoptotic effects of JQ1 can be increased in combination therapy 19 (this study). We showed that in mice, a less frequent application of JQ1 + romidepsin reduces tumour burden with same efficacy as JQ1 alone.

How do these results translate to clinical application? Based on different sensitivities of seminomas and ECs to JQ1, our results suggest that JQ1 is a potent treatment option for ECs and at higher doses also seminomas. While Nettersheim *et al*. previously demonstrated that romidepsin serves as a suitable treatment option for TGCTs in general 19, a combination of JQ1 + romidepsin allows for lower dosing and a less frequent application regimen. Clinical trials will reveal, whether the combination of JQ1 + romidepsin is suitable for treatment of (mixed) TGCTs and is well tolerated by the subject.

## Conflicts of interest

The authors declare no conflict of interest.

## Authors’ contributions

SJ performed experiments, analysed the data and wrote the manuscript. DN and HS interpreted the data, designed and financed the research study and wrote the manuscript. MF, SS and FH performed experiments. VS and MG contributed essential reagents and tools. GK contributed to writing and interpretation of data.

## Supporting information


**Figure S1** (A) *BRD2, 3, 4* and *T* expression in human TGCT cell lines (TCam‐2, NCCIT, 2102EP), Sertoli cells (FS1) and human fibroblasts (MPAF) as determined by expression microarray analysis. (B) Quantitation of BRD2, BRD3 and BRD4 protein levels in TGCT cell lines (TCam‐2, NCCIT, NT2/D1, 2102EP) and Sertoli cells (FS1) relative to HDAC1 levels. Western blot raw data is given in Figure 1B.

**Figure S2** (A) Cell morphology of JQ1 treated TGCT cell lines after 72 hrs of treatment. Scale bars: 250 μm. (B) AnnexinV/7AAD FACS analysis of apoptosis in 100 nM JQ1 treated TCam‐2 after 24 hrs, 8 days and 15 days. (C) Morphology of JQ1 treated TCam‐2 cells after 2, 4 and 16 weeks. Scale bar: 250 μm. Standard deviations, calculated by two‐tailed Student's *t*‐test, are given above each bar.

**Figure S3** (A) Cell morphology of FS1 and MPAF after 72 hrs of JQ1 treatment. Scale bars: 250 μm. (B) PI‐FACS analysis of cell cycle distribution of 100 nM JQ1 treated TCam‐2 after 16 weeks. Standard deviations, calculated by two‐tailed Student's *t*‐test, are given above each bar.

**Figure S4** (A, B) STRING‐based protein interaction prediction of genes upregulated (A) or downregulated (B) after 24 hrs of JQ1 treatment in NCCIT and TCam‐2 cells.

**Figure S5** (A) qRT‐PCR validation of genes found to be deregulated in TGCT cell lines after 72 hrs of 100 nM JQ1 treatment as determined by expression microarray analysis. (B) Expression levels of deregulated genes in JQ1 treated 2102EP at higher JQ1 concentrations (750–1250 nM) as determined by qRT‐PCR analysis. Standard deviations, calculated by two‐tailed Student's *t*‐test, are given above each bar.

**Figure S6** (A) *MYC* expression in JQ1 treated TCam‐2, NCCIT, NT2/D1 and 2102EP after 24 and 72 hrs of treatment as determined by qRT‐PCR analysis. (B) MYC protein levels in JQ1 treated TGCT cell lines after 24 and 72 hrs of treatment as determined by Western blot analysis. Standard deviations, calculated by two‐tailed Student's *t*‐test, are given above each bar.

**Data S1** (A‐D) Microarray analysis of JQ1‐treated TCam‐2 (A), NCCIT (B), FS1 (C) and MPAF (D) cells after 24 and 72 hrs normalized to solvent controls. Genes commonly deregulated after 24 and 72 hrs in the respective cell lines are listed in Venn diagrams. (E) Venn diagram depicting genes commonly deregulated in TCam‐2, NCCIT, FS1 and MPAF after 24 or 72 hrs JQ1 treatment. (F) GeneTrail analysis of genes upregulated in NCCIT after 72 hrs of JQ1 treatment. Enriched biological processes were further classified as ‘Endoderm’, ‘Mesoderm‘ ‘Ectoderm’, ‘Embryogenesis/Development’ and ‘Miscellaneous’ (Misc.).
