# Supplementary figures and images for: The bromodomain inhibitor JQ1 triggers growth arrest and apoptosis in testicular germ cell tumours in vitro and in vivo
Source: J Cell Mol Med. 2016 Dec 27;21(7):1300–14. doi: 10.1111/jcmm.13059 (PMC5487916; doi:10.1111/jcmm.13059)

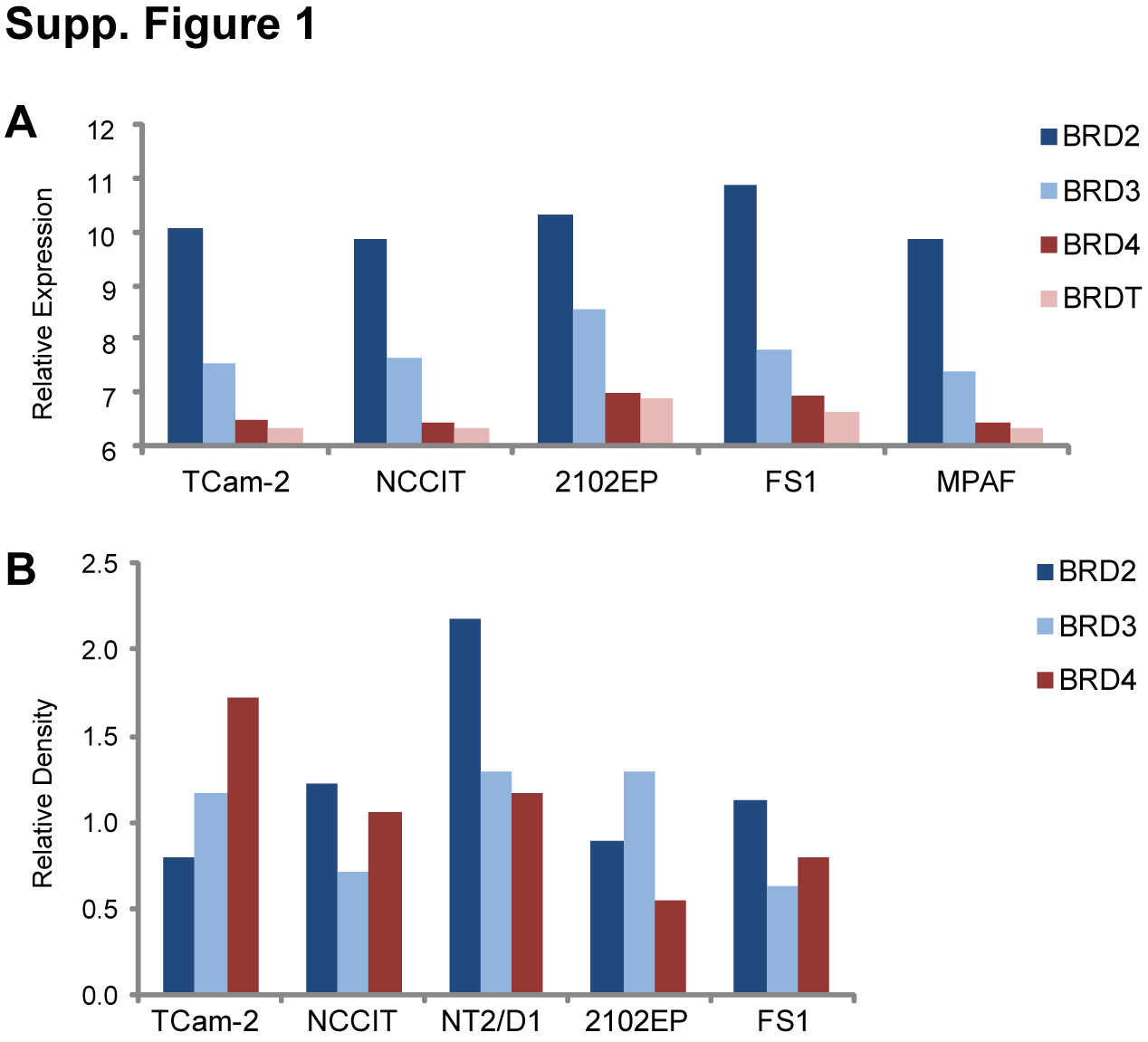

Supplement: Supplementary file 1 — Figure S1 (A) BRD2, 3, 4 and T expression in human TGCT cell lines (TCam‐2, NCCIT, 2102EP), Sertoli cells (FS1) and human fibroblasts (MPAF) as determined by expression microarray analysis. (B) Quantitation of BRD2, BRD3 and BRD4 protein levels in TGCT cell lines (TCam‐2, NCCIT, NT2/D1, 2102EP) and Sertoli cells (FS1) relative to HDAC1 levels. Western blot raw data is given in Figure 1B. [file JCMM-21-1300-s001.tif]

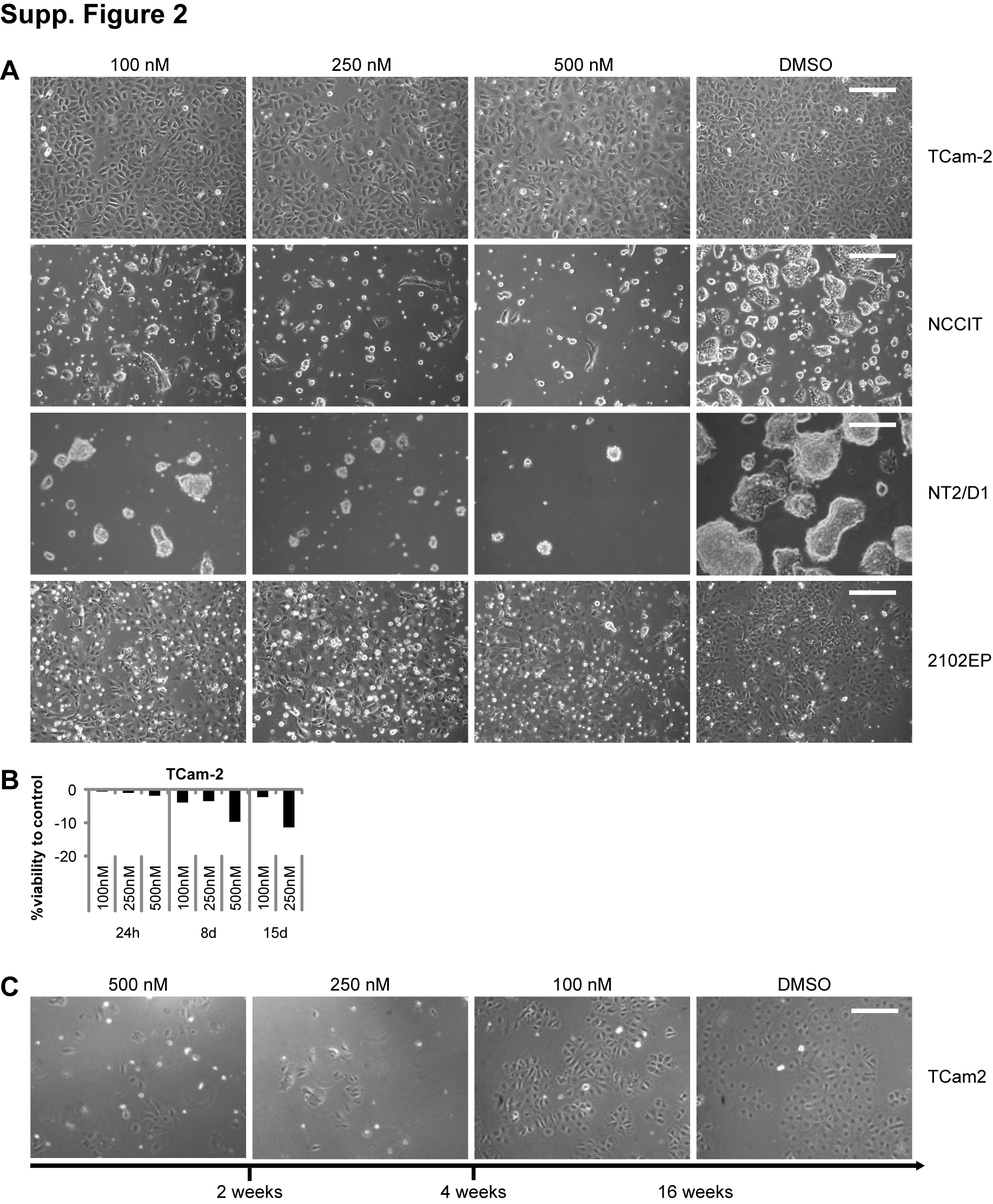

Supplement: Supplementary file 2 — Figure S2 (A) Cell morphology of JQ1 treated TGCT cell lines after 72 hrs of treatment. Scale bars: 250 μm. (B) AnnexinV/7AAD FACS analysis of apoptosis in 100 nM JQ1 treated TCam‐2 after 24 hrs, 8 days and 15 days. (C) Morphology of JQ1 treated TCam‐2 cells after 2, 4 and 16 weeks. Scale bar: 250 μm. Standard deviations, calculated by two‐tailed Student's t‐test, are given above each bar. [file JCMM-21-1300-s002.tif]

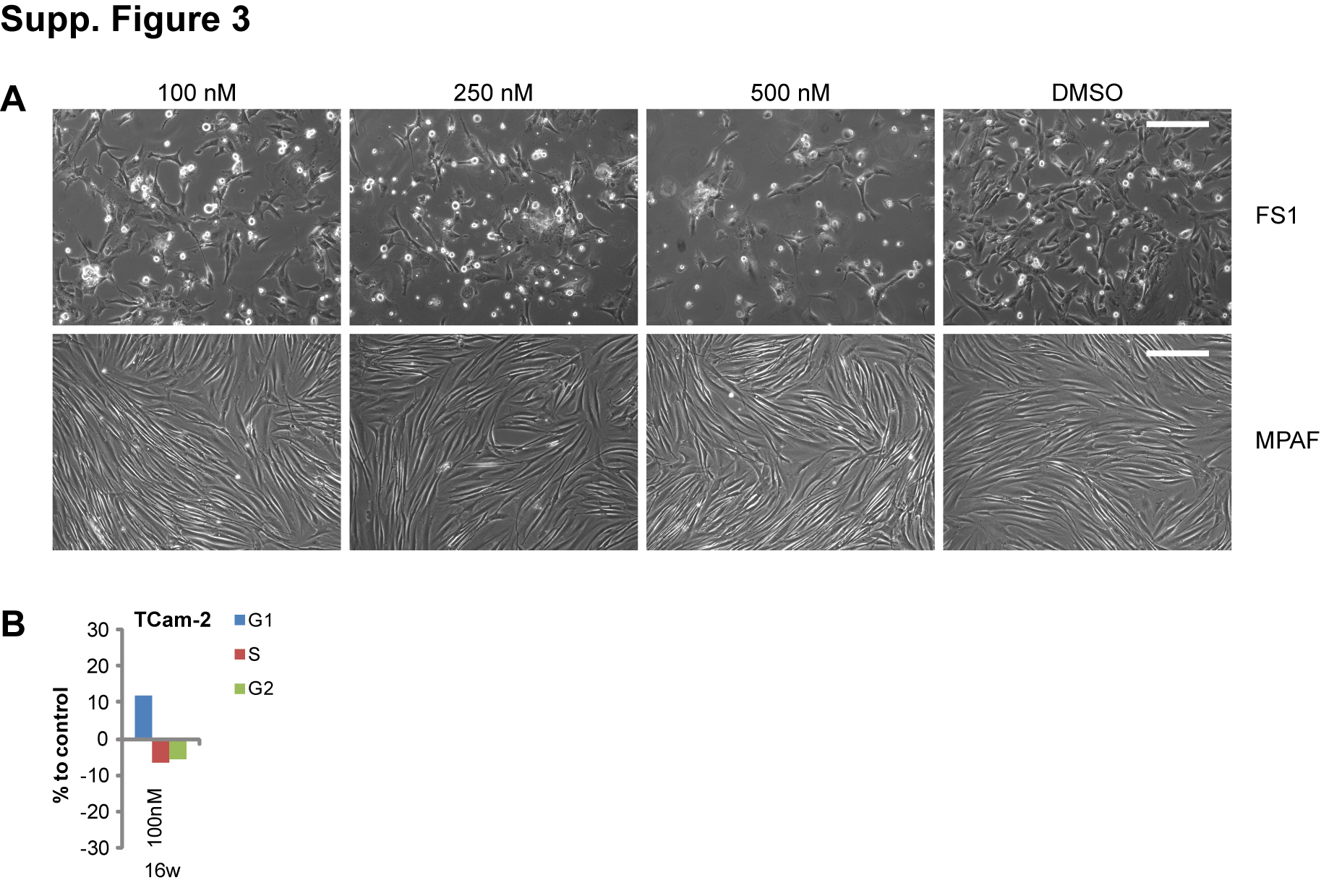

Supplement: Supplementary file 3 — Figure S3 (A) Cell morphology of FS1 and MPAF after 72 hrs of JQ1 treatment. Scale bars: 250 μm. (B) PI‐FACS analysis of cell cycle distribution of 100 nM JQ1 treated TCam‐2 after 16 weeks. Standard deviations, calculated by two‐tailed Student's t‐test, are given above each bar. [file JCMM-21-1300-s003.tif]

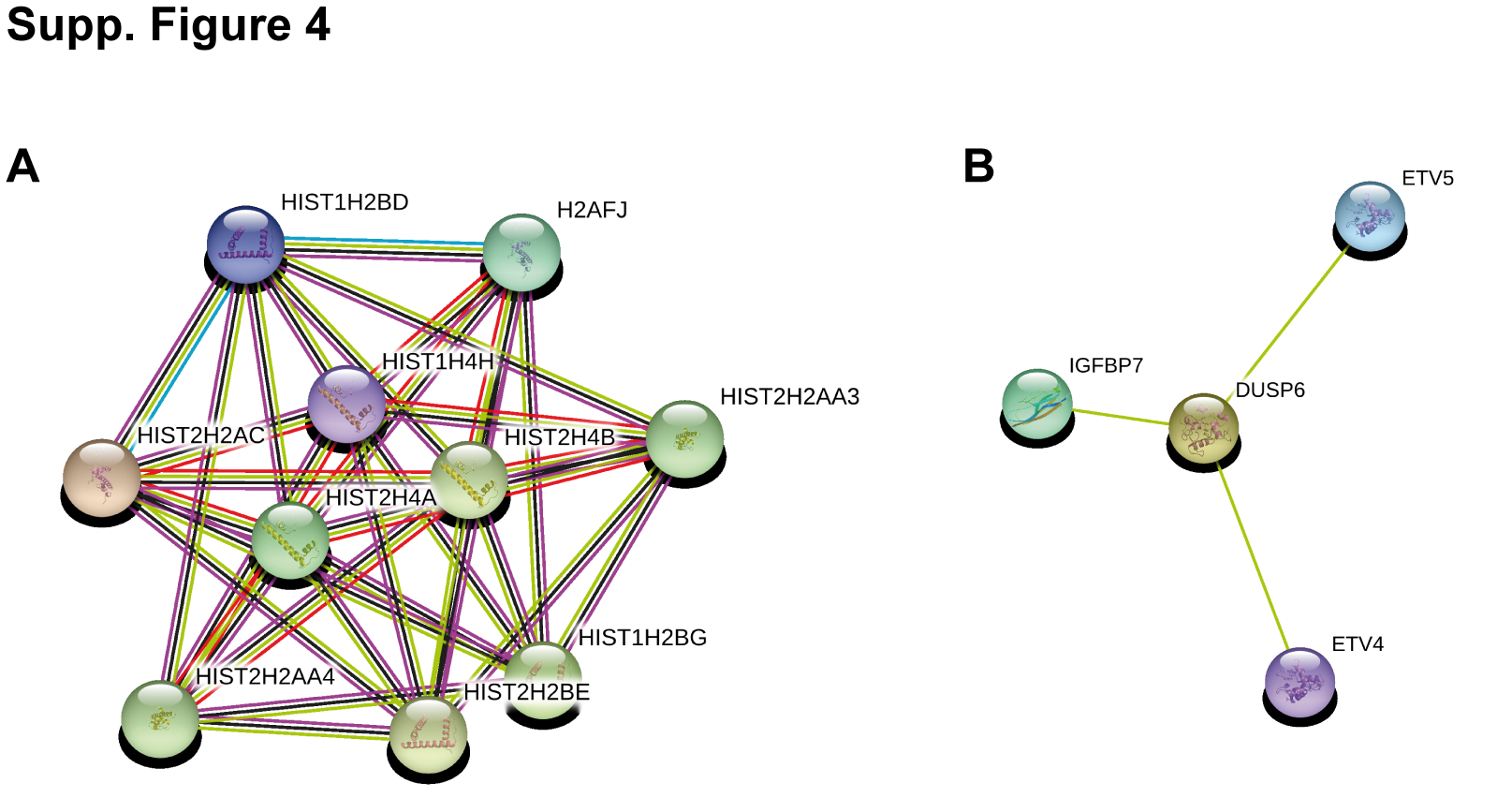

Supplement: Supplementary file 4 — Figure S4 (A, B) STRING‐based protein interaction prediction of genes upregulated (A) or downregulated (B) after 24 hrs of JQ1 treatment in NCCIT and TCam‐2 cells. [file JCMM-21-1300-s004.tif]

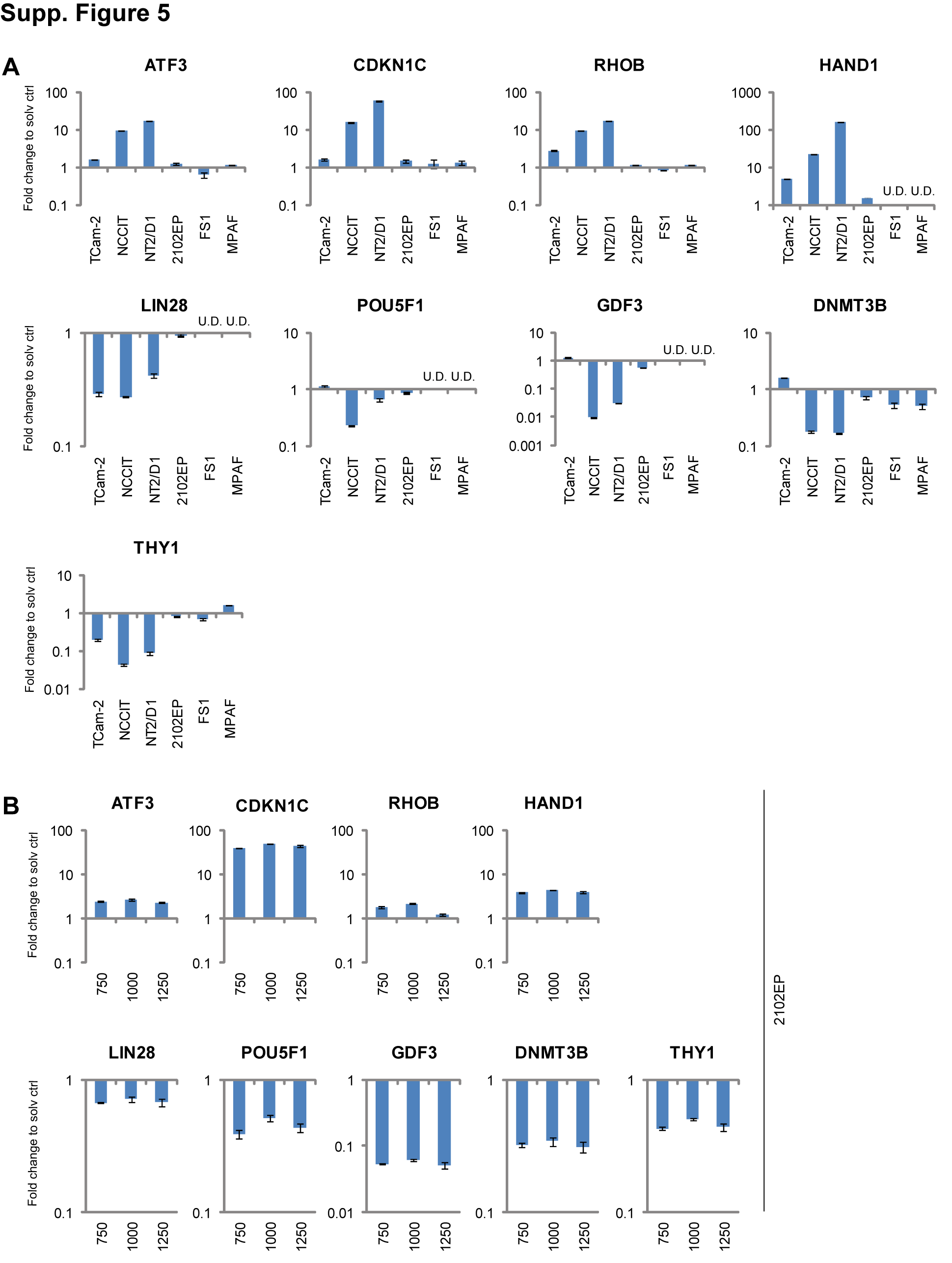

Supplement: Supplementary file 5 — Figure S5 (A) qRT‐PCR validation of genes found to be deregulated in TGCT cell lines after 72 hrs of 100 nM JQ1 treatment as determined by expression microarray analysis. (B) Expression levels of deregulated genes in JQ1 treated 2102EP at higher JQ1 concentrations (750–1250 nM) as determined by qRT‐PCR analysis. Standard deviations, calculated by two‐tailed Student's t‐test, are given above each bar. [file JCMM-21-1300-s005.tif]

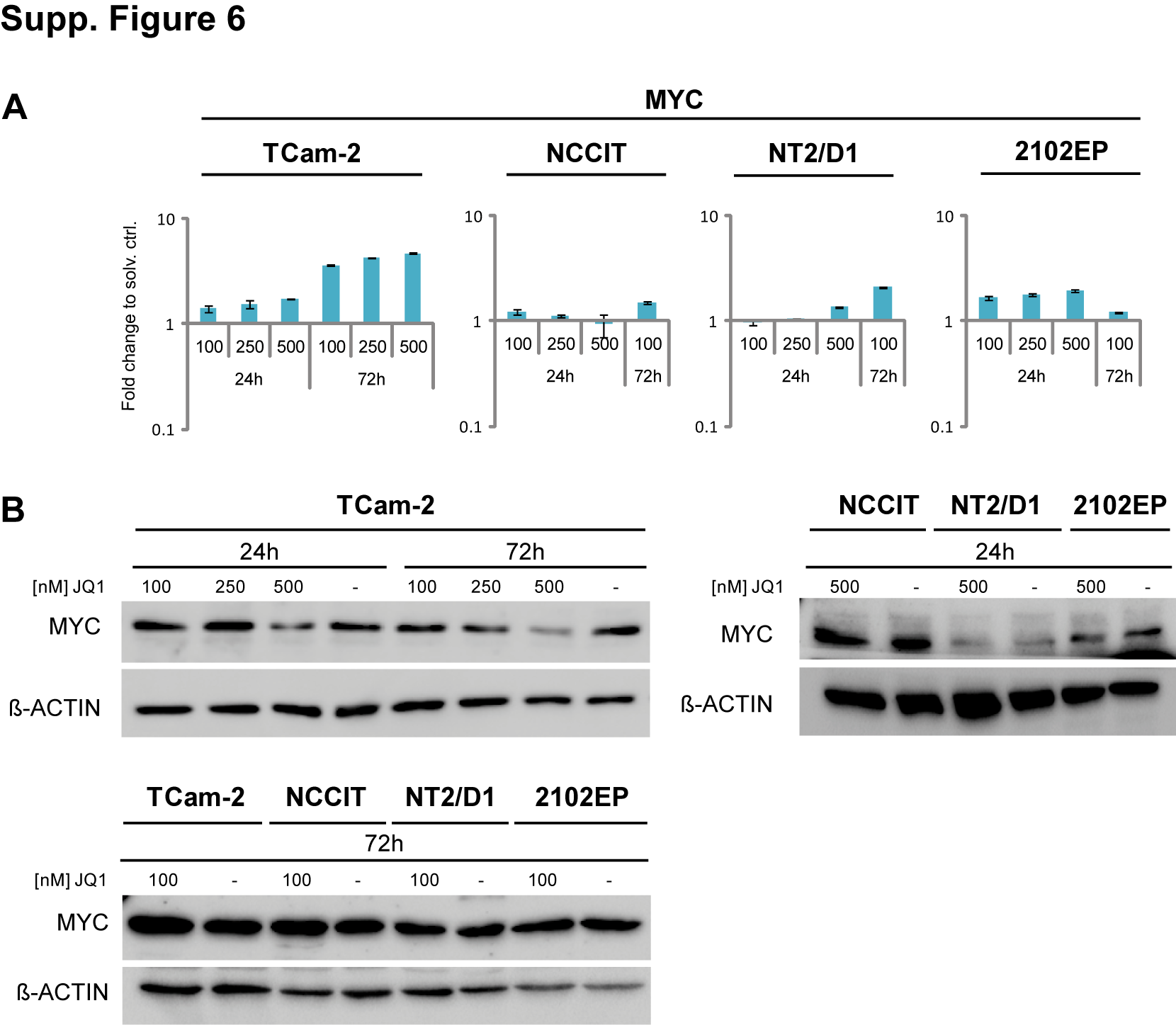

Supplement: Supplementary file 6 — Figure S6 (A) MYC expression in JQ1 treated TCam‐2, NCCIT, NT2/D1 and 2102EP after 24 and 72 hrs of treatment as determined by qRT‐PCR analysis. (B) MYC protein levels in JQ1 treated TGCT cell lines after 24 and 72 hrs of treatment as determined by Western blot analysis. Standard deviations, calculated by two‐tailed Student's t‐test, are given above each bar. [file JCMM-21-1300-s006.tif]
